# Musculoskeletal Disorders in Elderly Patients With Diabetes Mellitus in a Rural Community of Bangladesh

**DOI:** 10.7759/cureus.84139

**Published:** 2025-05-14

**Authors:** Mohammad Ferdous Ur Rahaman, Jannatara Shefa, Chowdhury Adnan Sami, Mahbubur Rahman, Abu Kamran Rahul

**Affiliations:** 1 Internal Medicine, Bangabandhu Sheikh Mujib Medical University, Dhaka, BGD; 2 Public Health, Bangabandhu Sheikh Mujib Medical University, Dhaka, BGD

**Keywords:** chronic pain, diabetes mellitus, elderly population, functional disability, musculoskeletal disorders, obesity

## Abstract

Background

Among the elderly population of Bangladesh, diabetes mellitus (DM) is highly prevalent. DM has a strong relationship with musculoskeletal (MSK) disorders responsible for chronic pain, disability, and morbidity. Nevertheless, MSK disorder data of elderly diabetics are scarce in the literature, particularly in rural setups. The objective of this study was to evaluate the status of MSK disorders and their associated factors among elderly diabetic people living in rural Bangladesh.

Methods

In this cross-sectional study, we included participants with diabetes over 60 years from six rural villages in Muksudpur Union, Bangladesh. The details of the study, including data collection using the Community-Oriented Program for the Control of Rheumatic Diseases (COPCORD) questionnaire, followed clinical examination. Disability was assessed using the Health Assessment Questionnaire (HAQ), and associated factors were analyzed statistically.

Results

A total of 234 older adults with diabetes were included from six villages of rural Muksudpur Union, Bangladesh, and were recruited. They were also subjected to clinical examinations after completing the COPCORD questionnaire. Disability was measured by the HAQ and analyzed by stepwise multiple linear regression. A total of 234 persons participated, yielding 179 (76.5%) with MSK pain, of whom 144 (61.5%) were women and 90 (38.5%) men. The lower back (50 (28.6%)), knee (38 (21.7%)), and foot (23 (13.1%)) were the most reported pain sites. MSK disorders were also significantly associated with obesity (BMI ≥ 25 kg/m²) and uncontrolled diabetes. Functional disability was remarkable; 130 (55.5%) of participants were moderately disabled; 71 (30.3%), mildly disabled; and 33 (14.1%), severely disabled.

Conclusion

This study highlighted the high burden of MSK pain among elderly diabetics in rural Bangladesh, with women, obesity, and poor glycemic control as significant predictors. This finding emphasizes the need for early screening and weight control and for integrated healthcare strategies to ameliorate mobility and life quality in this susceptible population.

## Introduction

The demographics of the country are changing dramatically, with the population of older people in Bangladesh growing rapidly. It is expected to rank in the top 10 countries with the most significant number of elderly citizens over the coming decades. A study indicates that by 2050, approximately 22.3% of Bangladesh’s population will be aged 60 or older, a significant rise from 8.1% in 2020 [1]. As reported by the United Nations (UN), the older population aged 60 and older is projected to triple over the next 40 years and will account for more than 20% of the global population by 2050 [2]. By then, one in five of the elderly will also be more than 80 years old. This growing elderly population is most notable because of a higher life expectancy, especially in developing countries.

Musculoskeletal (MSK) disorders have been recognized by the World Health Organization (WHO) as one of the most significant contributors to disability in older age and one of the most important health challenges for aging populations worldwide [3]. Diabetes mellitus (DM) is also becoming increasingly important in the global public health scene. In 2017, there were approximately 451 million people across the globe (aged 18-99) suffering from diabetes, which is estimated to increase to 693 million by the year 2045 [4]. Apart from being one of the leading causes of death and disability, diabetes is also a significant cause of economic loss and a global public health problem. Bangladesh has the second highest prevalence of diabetes in Southeast Asia among adults 20-79 years of age. In 2013, 5.1 million people were living in Bangladesh with diabetes, and this number is projected to rise to 8.2 million by 2035 [2].

There are different MSK disorders strongly associated with diabetes, which lead to chronic pain, impaired mobility, and disability. The burden of four significant MSK conditions; osteoarthritis (OA), rheumatoid arthritis (RA), osteoporosis (OP), and low back pain (LBP) has been increasing, and the global disease burden 2021 study provides evidence that MSK conditions remain a leading cause of disability worldwide, with significant variations across regions and populations [5]. The results highlighted how rampant MSK diseases have become due to an aging population with longer life expectancies, thus having a serious toll on health outcomes and quality of life. As the older population continues to grow, healthcare practitioners need to understand the burden of MSK disorders among the elderly.

Nevertheless, geriatric muscular disorders are still an understudied area [6], and most of the studies primarily focus on healthcare accessibility in urban centers rather than rural ones [7]. The unique needs of older adults with MSK conditions are often neglected in medical education and healthcare systems, resulting in a lack of awareness, diagnosis, and management of MSK conditions. Elderly diabetic patients are prone to many other conditions, including MSK disorders, which are also increasing in many low- and middle-income countries (LMICs) such as Bangladesh. These insights could inform effective healthcare strategies, policies, and interventions that improve quality of life while potentially reducing the wider economic and social impacts.

This study aimed to determine the prevalence, anatomical distribution, and severity of MSK disorders and to identify key demographic and metabolic factors, including obesity and glycemic control, associated with MSK pain and disability among elderly individuals with diabetes in rural Bangladesh.

## Materials and methods

Study design and setting

To determine the prevalence of MSK pain among elderly persons with DM in a rural community of Bangladesh, a descriptive cross-sectional study was conducted. The participants included patients aged 60 and older who were diagnosed with DM. This study was conducted from January to December 2023 in six randomly selected villages of Muksudpur Union, Dohar Upazila, Dhaka District, Bangladesh.

Study population and sampling

In this backdrop, the study was conducted in the Dohar Upazila (51.4 km from Dhaka city), which is suitable for the site of the study because of greater socioeconomic representation and accessibility. A purposive cluster sampling strategy was employed here. These include eight unions with a community clinic as a primary healthcare setting. The total population of 20,436 in 19 villages of Muksudpur union is being served by only one community clinic.

Data collection process

Data were collected in three phases through the study stages as follows: identification of participants using Community-Oriented Program for the Control of Rheumatic Diseases (COPCORD) conversations to identify individuals with DM and MSK pain, with details of symptoms, functional limitations, and demographic details were obtained. Second, information was collected on the characteristics of the household, sociodemographic, and socioeconomic factors. Third, a structured history-taking process and physical examination were performed by trained physicians using the COPCORD examination sheet. Muksudpur Community Clinic was chosen as the COPCORD clinic where newly and previously diagnosed elderly diabetic patients were assessed after receiving administrative approval (questionnaires included in the appendices).

Training and data collection personnel

A team of six trained research field workers, assisted by Family Welfare Assistants (FWAs) and Health Assistants (HAs), conducted the door-to-door survey. Willing participants were assigned a unique COPCORD ID. The data collectors, trained by authors from the Internal Medicine Department at BSMMU, administered the validated Bengali version of the COPCORD questionnaire to identify individuals with diabetes and MSK disorders. Participants who screened positive were scheduled for clinical evaluation at the COPCORD clinic. At the clinic, detailed interviews and physical examinations were conducted to assess MSK pain severity, disability, and functional limitations. All data were carefully reviewed by the lead author to ensure accuracy and consistency.

Operational definitions

Positive Respondent

A participant was considered positive for MSK pain if they reported experiencing pain or discomfort in muscles, bones, joints, or any part of the body within the preceding week. Individuals whose MSK pain had appeared, worsened, or disappeared in the previous week were also categorised as positive respondents.

Disability Classification

Disability was assessed using the validated Bengali version of the Health Assessment Questionnaire (B-HAQ) [6]. Based on HAQ scores, disability was classified into three categories: mild disability (0.1-1.0), moderate disability (1.1-2.0), and severe disability (2.1-3.0) [7].

Diabetes Definition

Participants were considered diabetic if their glycated hemoglobin was >6.4%, or if they self-reported being previously diagnosed with diabetes and were on anti-diabetic medication, which was confirmed by the research physicians [8].

Pain Severity

The severity of MSK pain was classified using a 10-point Visual Analog Scale (VAS), ranging from mild (1-3) to moderate (4-6) to severe (7-10) [9].

The COPCORD questionnaire, which had been translated into Bengali and previously validated for use in Bangladeshi populations by Haq et al. [10]. Functional disability was assessed using the B-HAQ, whose cross-cultural adaptation and psychometric validation were conducted by Islam et al. [6]. The B-HAQ demonstrated good internal consistency (Cronbach’s α = 0.89) and test-retest reliability (intraclass correlation coefficient = 0.93), confirming its suitability for use in elderly populations with MSK complaints in Bangladesh.

Statistical analysis

The data were analyzed using IBM SPSS Statistics software version 26 (IBM Corp., Armonk, NY). Frequency distributions and percentages were calculated for categorical variables, and the mean (±SD) was used for numerical variables. Frequency rates for MSK pain were estimated along with 95% confidence intervals by the Wilson method. Categorical associations (sex, BMI category, HbA1c, and smoking status) with the presence of MSK pain were tested using the chi-square test. The categories of disability, as evaluated by the B-HAQ, were compared according to demographic and clinical variables. A p<0.05 was regarded as statistically significant.

Ethical considerations

Ethical approval for the study was obtained from the Institutional Review Board (IRB) of Bangabandhu Sheikh Mujib Medical University (BSMMU/2023/2719). The purpose and procedures of the study were clearly explained to all participants, and written informed consent was obtained before their inclusion. Participants were assured confidentiality, and no additional burden was imposed on them. Potential risks were carefully assessed, and participants had the right to withdraw from the study at any point.

## Results

Socio-demographic characteristics of the study population

A total of 234 elderly individuals (aged ≥60) were enrolled. Among the 234 participants, 179 (76.5%; 95% CI: 70.7%-81.5%) reported MSK pain. Women comprised 144 individuals (61.5%; 95% CI: 55.2%-67.5%) and men 90 individuals (38.5%; 95% CI: 32.5%-44.8%) of the study population. Regarding age distribution, 203 (86.8%) participants were between 60 and 70 years of age, and 31 (13.2%) participants were above 70 years. In terms of educational attainment, 116 (49.6%) participants were illiterate, 64 (27.3%) had completed primary education, 48 (20.5%) completed secondary education, and six (2.6%) achieved higher secondary or above. Regarding occupation, homemakers comprised 111 (47.4%), service holders 41 (17.5%), businesspersons 10 (4.3%), agricultural workers six (2.6%), and others (including retired individuals) 64 (27.4%). In terms of family income, 131 (56.0%) had a monthly income below 20,000 BDT, 90 (38.5%) had between 20,000 and 39,000 BDT, and 13 (5.6%) had income greater than 40,000 BDT (Table 1).

**Table 1:** Socio-demographic characteristics of the study population (n=234) BMI: body mass index, HbA1c: glycated hemoglobin, BDT: Bangladeshi Taka

Variables	Frequency (n)	Percentage (%)
Sex		
Male	90	38.5
Female	144	61.5
Age		
60–70 years	203	86.8
>70 years	31	13.2
Education		
Illiterate	116	49.6
Primary	64	27.3
Secondary	48	20.5
Higher secondary and above	6	2.6
Occupation		
Agriculture	6	2.6
Business	10	4.3
Service holder	41	17.5
Homemaker	111	47.4
Others (including retired)	64	27.4
Family’s monthly income		
<20,000 BDT	131	56.0
20,000–39,000 BDT	90	38.5
>40,000 BDT	13	5.6
Prevalence of musculoskeletal pain	179	76.5
HbA1c status		
≤6.4%	109	46.6
>6.4%	125	53.4
BMI status		
18.5–24.9	132	56.4
25–29.9	80	34.2
≥30	22	9.4

Prevalence of MSK disorders across age groups

Among participants with MSK pain, the highest prevalence was noted in the 60 years age group (80 (44.7%)), followed by the 61-70 years group (73 (40.8%)) and those older than 71 years (26 (14.5%)) (Table 2).

**Table 2:** Musculoskeletal disorders in different age groups (n=179)

Age group (years)	Frequency (n)	Percentage (%)
60	80	44.7
61–70	73	40.8
>71	26	14.5
Total	179	100.0

Distribution of MSK pain by body region

Out of the 179 participants reporting MSK pain, 175 specified the affected anatomical sites. The most commonly affected sites were the lower back (50 (28.6%)), knee (38 (21.7%)), and foot (23 (13.1%)). Other reported sites included the shoulder (14 (8.0%)), ankle (11 (6.3%)), thigh (10 (5.7%)), arm (seven (4.0%)), forearm (seven (4.0%)), hip (five (2.9%)), neck (four (2.3%)), chest (three (1.7%)), and elbow (three (1.7%)) (Table 3).

**Table 3:** Pain in different parts of the body (n=175)

Pain site	Frequency (n)	Percentage (%)
Ankle joint	11	6.3
Chest	3	1.7
Elbow joint	3	1.7
Arm	7	4.0
Foot	23	13.1
Forearm	7	4.0
Hip	5	2.9
Knee	38	21.7
Lower back	50	28.6
Neck	4	2.3
Thigh	10	5.7
Shoulder	14	8.0

Severity of MSK pain

Regarding pain severity assessed by the VAS score, mild pain (VAS score 0-3) was reported by 155 (86.6%) participants, moderate pain (VAS score 4-6) by 18 (10.1%), and severe pain (VAS score 7-10) by six (3.4%) participants (Table 4).

**Table 4:** Severity of musculoskeletal pain (n=179)

Severity category (VAS score)	Frequency (n)	Percentage (%)
Mild (0–3)	155	86.6
Moderate (4–6)	18	10.1
Severe (7–10)	6	3.4

Association between diabetes, smoking, and MSK disorders

Diabetes was present in 109 (46.6%) participants with MSK pain, compared to 70 (29.9%) without MSK pain (p = 0.024). Smoking was reported by 116 (49.6%) of participants with MSK pain and by 63 (26.9%) without MSK pain, indicating a statistically significant association (p = 0.001) (Table 5).

**Table 5:** Musculoskeletal disorders among diabetes and smoker patients (n=234) MSK: Musculoskeletal pain

Trait	Status	MSK pain (Yes)	MSK pain (No)	P-value
Diabetes	Present	109 (46.6%)	70 (29.9%)	0.024
Smoking	Smoker	116 (49.6%)	63 (26.9%)	0.001

Metabolic and anthropometric parameters

Poor glycemic control (HbA1c >6.4%) was identified in 125 (53.4%) participants. Regarding BMI, 132 (56.4%) had normal BMI (18.5-24.9 kg/m²), 80 (34.2%) were overweight (25-29.9 kg/m²), and 22 (9.4%) were obese (≥30 kg/m²) (Table 1).

Disability status based on HAQ score

According to the HAQ scores, mild disability (HAQ 0-1) was found in 71 (30.3%) participants, moderate disability (HAQ 1.1-2.0) in 130 (55.6%), and severe disability (HAQ 2.1-3.0) in 33 (14.1%) participants (Table 6).

**Table 6:** Disability classification based on HAQ score (n=234) HAQ: Health Assessment Questionnaire

Disability category	Frequency (n)	Percentage (%)
Mild (HAQ 0–1)	71	30.3
Moderate (HAQ 1.1–2.0)	130	55.6
Severe (HAQ 2.1–3.0)	33	14.1

## Discussion

Managing disabling MSK pain is one of the most prevalent health issues faced in clinical practice, with millions of individuals suffering from it all over the world. Throughout history, human civilization has faced and will continue to face this global health issue that burdens the individual and society physically, emotionally, and economically [11,12]. MSK disorders are a ubiquitous, neglected global health problem with widespread disability and cost, but this is not matched by effective research and education to motivate preventive and therapeutic interventions. This study aspired to explore the frequency of MSK pain in elderly diabetics who are known to be a vulnerable group to such morbidity.

Prevalence and gender disparity in MSK pain

A higher proportion of elderly individuals with diabetes suffer from MSK pain (76.5%). Among them, the prevalence of MSK pain appears to be more common in women (61.5%) than in men (38.5%). This pattern is consistent with a similar study performed in rural Dibrugarh, India, in which 50.67% of the elderly experienced MSK disorders [13]. Likewise, in 2005, data from the COPCORD study revealed higher proportions of men (48%) and women (65%) with MSK diseases, reaffirming the observed data of irrefutably higher impact on females [14]. There are several reasons for this gender imbalance. Women of all socioeconomic backgrounds can often be seen to be engaged in heavy duties like housekeeping, gardening, and taking care of their sick family members, which all lead to increased incidences of MSK disorders. Other common contributors are bad posture, prolonged squatting, heavy weightlifting, anxiety, deficient peer support, and poor mental health. Work that requires recurrent kneeling and squatting has also been associated with a greater risk of MSK disorders [10].

Aging and the risk of MSK disorders

Aging is a significant risk factor for MSK disorders, and the burden of MSK conditions has shown an increase with age in the prevalence of MSK pain. Our findings corroborate those of earlier studies in Bangladesh that also found an increased prevalence of skeletal muscle (MSK) disorders among the elderly. Degenerative joint diseases are projected to be one of the top causes of disability by 2040 due to the increasing age of the population and rising life expectancies [15].

Association between diabetes and MSK disorders

Obesity (BMI ≥ 25 kg/m²) was also significantly linked to MSK discomfort. Compared to those with a normal BMI, pain complaints in the lower legs and knees were significantly more pronounced for overweight and obese individuals, while pain in the arms and joints was significantly more prominent in obese individuals [16]. Due to the close association of diabetes, obesity, and MSK health, lifestyle changes such as weight reduction and physical activity are essential to attenuate MSK burden among elderly diabetics.

Our findings highlight a significant association between diabetes and MSK pain. MSK pain is common, but little is known about its associations with diabetes. Patients with diabetes had a higher prevalence of MSK pain compared to non-diabetic individuals, thus further highlighting the contribution of metabolic dysfunction to rheumatologic diseases. An observational study report found that diabetic patients had a higher risk of frozen shoulder, carpal tunnel syndrome, and trigger finger, supporting the theory that chronic hyperglycemia may lead to connective tissue and joint changes [17].

In our study, the knee joint and the lower back were the most prevalent sites of pain (22.4% and 27.9%, respectively), which is consistent with findings reported in aging populations globally. Obesity, bad posture, heavy lifting, and lack of physical activity are among the many causes of lower back pain (LBP) [18]. So, chronic back pain is difficult and often accompanied by age-related degenerative changes to the structures of the spine. Older age is an independent risk factor for the development of LBP [19], with those aged >35 years at a nine times increased risk of LBP than younger people [19].

Potential role of vitamin D deficiency

One possible risk factor could be the combined malnourishment status and vitamin D deficiency in low-resource settings due to lack of sunlight exposure, leading to vitamin D deficiency and factors related to the economy [20]. Vitamin D deficiency may play a contributory role in chronic MSK pain among elderly individuals in Bangladesh. Several studies have linked hypovitaminosis D to diffuse MSK pain, sarcopenia, and impaired postural stability [21,22].

Impact of MSK pain on functional disability

MSK disorders are a major source of loss of functional capabilities apart from pain. Disability was assessed using the HAQ; 55.6% of the participants had moderate disability, 30.3% mild disability, and 14.1% severe disability. MSK pain was also shown to make the elderly lose both mobility and independence, as these findings highlight. This can cause decreased physical activity, social isolation, and ultimately a deterioration of quality of life, all of which highlight the importance of more complete pain management approaches.

Strengths and limitations

This study uniquely addresses MSK disorders in elderly diabetics in rural Bangladesh using validated tools (Bengali COPCORD and B-HAQ) and clinical confirmation by trained physicians. Its community-based design and focus on an underserved population enhance its relevance to rural health policy and primary care.

This study has several limitations. First, the relatively small sample size of 234 participants, drawn from a single union (Muksudpur, Dohar Upazila, Dhaka District), may limit the generalizability of the findings to the broader elderly diabetic population of Bangladesh. Second, voluntary participation may have excluded individuals with severe mobility impairment, cognitive decline, or multiple comorbidities, potentially underestimating the true burden of MSK disorders. Third, the reliance on self-reported data through the COPCORD questionnaire introduces recall bias, possibly leading to over- or underestimation of symptom severity. Additionally, the study’s cross-sectional design inherently limits the ability to draw causal inferences between diabetes, obesity, glycemic control, and MSK disorders. While significant associations were identified, the temporal relationship between risk factors and MSK outcomes cannot be confirmed. To strengthen the evidence base, future longitudinal cohort studies are recommended. Finally, the absence of a non-diabetic control group and the lack of assessment of factors such as dietary habits, vitamin D status, and environmental exposures may limit the comprehensiveness of the findings.

## Conclusions

This study demonstrates the substantial burden of MSK pain among older-aged diabetics in rural Bangladesh. There was a higher incidence in women, and complaints were mainly LBP and knee joint pain. The strong relationship between diabetes, obesity, and MSK pain calls for integrated management strategies including diabetes control, weight management, and MSK rehabilitation. Functional impairment had been severe, limiting daily tasks and quality of life. These findings strongly recommend early screening with physiotherapy and lifestyle modification to reduce the disability and increase mobility. At the policy level, these findings support incorporating geriatric MSK care into Bangladesh’s Essential Service Package and strengthening the capacity of rural community clinics to address functional disability in aging populations. Though the small sample size and reliance on self-reported data are limitations, the study highlights the pressing need for better MSK disorder management in older diabetic patients. With the aging population of Bangladesh poised to expand rapidly, evidence-based policies will be important to improve mobility and overall well-being among this at-risk aging population.

## Appendices

Appendix 1

**Table 7:** Modified COPCORD (survey information) COPCORD: Community-Oriented Program for Control of Rheumatic Diseases

Variable code	Variable	Response
AA1	Primary sampling unit (PSU)	No
AA2	Household	No
AA3	Household status (1. Male, 2. Female)	
AA4	Name of participant	
AA5	Age of participant	
AA6	Telephone/mobile number of participant	

**Table 8:** Modified COPCORD (articular profile) COPCORD: Community-Oriented Program for Control of Rheumatic Diseases

Variable code	Variable	Response
CC1	Onset of symptoms (1. Acute. 2. Subacute. 3. Chronic)	
CC2	Duration (1. Less than 6 weeks. 2. More than 6 weeks)	
CC3	Pattern (1. Monoarticular. 2. Oligoarticular. 3. Polyarticular)	
CC3.1	Symmetry of involvement (1. Symmetrical. 2. Asymmetrical)	
CC4	Course of disease (1. Persistent. 2. Recurrent. 3. Static remission. 4. Partial remission and relapse)	

**Table 9:** Modified COPCORD (back pain) COPCORD: Community-Oriented Program for Control of Rheumatic Diseases

Variable code	Site	Type	Duration
DD1	Back pain (0. No. 1. Inflammatory. 2. Mechanical)		
DD1.1	Duration (1. Less than 3 months. 2. More than 3 months)		

**Table 10:** Modified COPCORD (joint examination) COPCORD: Community-Oriented Program for Control of Rheumatic Diseases

Variable Code	Joint	Right	Left	Both	Swelling	Tenderness	Deformity	ROM Restriction
	Temporomandibular							
	Sterno-clavicular							
	Acromioclavicular							
	Shoulder							
	Elbow							
	Wrist							
	Interphalangeal 1							
	Distal Interphalangeal 2							
	Distal Interphalangeal 3							
	Distal Interphalangeal 4							
	Distal Interphalangeal 5							
	Proximal Interphalangeal 2							
	Proximal Interphalangeal 3							
	Proximal Interphalangeal 4							
	Proximal Interphalangeal 5							
	Metacarpophalangeal 1							
	Metacarpophalangeal 2							
	Metacarpophalangeal 3							
	Metacarpophalangeal 4							
	Metacarpophalangeal 5							
	Hip							
	Knee							
	Ankle							
	Midtarsal							
	Metatarsophalangeal 1							
	Metatarsophalangeal 2							
	Metatarsophalangeal 3							
	Metatarsophalangeal 4							
	Metatarsophalangeal 5							
	Sacroiliac Joint							

Appendix 2

**Table 11:** Health Assessment Questionnaire (HAQ) Instructions for participant: Please indicate your level of difficulty in performing the following activities over the past week.

Activity	Without any difficulty (0)	With some difficulty (1)	With much difficulty (2)	Unable to do (3)
Dressing and grooming yourself				
Rising from a chair				
Eating meals				
Walking outdoors on flat ground				
Getting in and out of bed				
Bathing yourself				
Reaching and stretching arms above shoulder level				
Opening a new carton of milk				
Running errands and shopping				

Appendix 3

**Table 12:** Severity of pain: assessed by visual analog scale (VAS)

0	1	2	3	4	5	6	7	8	9	10
